# Prediction of pulmonary infection in patients with severe myelitis by NPAR combined with spinal cord lesion segments

**DOI:** 10.3389/fneur.2024.1364108

**Published:** 2024-02-28

**Authors:** Fan Yang, Ruirui Dong, Yating Wang, Junshuang Guo, Qiuling Zang, Lijun Wen, Peipei Huang, Jinjin Qin, Dandan Song, Zhiping Ren, Junfang Teng, Wang Miao

**Affiliations:** Neuro-Intensive Care Unit, The First Affiliated Hospital of Zhengzhou University, Zhengzhou, China

**Keywords:** severe myelitis, pulmonary infection, NPAR, high cervical cord lesion, nomogram

## Abstract

**Objectives:**

To investigate the risk factors of pulmonary infection in patients with severe myelitis and construct a prediction model.

**Methods:**

The clinical data of 177 patients with severe myelitis at admission from the First Affiliated Hospital of Zhengzhou University from January 2020 to December 2022 were retrospectively analyzed. The predicting factors associated with pulmonary infection were screened by multivariate logistic regression analysis, and the nomogram model was constructed, and the predictive efficiency of the model was evaluated, which was verified by calibration curve, Hosmer–Lemeshow goodness-of-fit test and decision curve analysis.

**Results:**

Of the 177 patients with severe myelitis, 38 (21.5%) had pulmonary infection. Multivariate logistic regression analysis showed that neutrophil percentage to albumin ratio (NPAR) (*OR* = 6.865, 95%*CI*:1.746–26.993, *p* = 0.006) and high cervical cord lesion (*OR* = 2.788, 95%*CI*:1.229–6.323, *p* = 0.014) were independent risk factors for pulmonary infection, and the combined nomogram could easily predict the occurrence of pulmonary infection, with a C-index of 0.766 (95% *CI*: 0.678–0.854). The calibration curve, Hosmer-Lemeshow goodness-of-fit test (*χ*^2^ = 9.539, *p* = 0.299) and decision curve analysis showed that the model had good consistency and clinical applicability.

**Conclusion:**

The nomogram model constructed based on NPAR combined with high cervical cord lesion at admission has good clinical application value in predicting pulmonary infection in patients with severe myelitis, which is conducive to clinicians’ evaluation of patients.

## Introduction

Acute myelitis is a neuroinflammatory disease involving the spinal cord, characterized by an acute onset, severe symptoms, and a very poor prognosis, with up to two-thirds of patients experiencing moderate to severe disability (1). Approximately 50% of patients suffer from paraplegia, which predisposes them to pulmonary dysfunction (2). In patients with severe myelitis, the incidence of pulmonary infection is even higher due to the severity of the condition and associated motor dysfunction. Pulmonary infections due to myelopathy have been found to occur in 24.6 to 35.5% (3, 4). Not only do pulmonary infections prolong hospital stays and increase medical costs (5), but they also serve as significant risk factors for mortality (6, 7). The occurrence of pulmonary infection can have an important effect on the prognosis of patients with myelitis. Therefore, early screening of high-risk groups for pulmonary infection, implementation of stratified management and active intervention have important clinical significance for improving patient prognosis and reducing disability.

In patients with acute myelitis, there are differences in the level and extent of spinal cord lesions, with thoracic spinal cord involvement being the most common, followed by cervical and lumbar spinal cords involvement. This difference in spinal cord lesion segments can affect the occurrence and severity of infection to some extent. As a neuroinflammatory disease, inflammation and immunity play an important role in the pathogenesis of acute myelitis. Several studies have found that inflammation and immune status are closely related to infection, disease severity and prognosis (3, 8–10). Decreased nutrition and metabolism tend to affect the body’s immunity, thereby increasing susceptibility to infection (11, 12). These factors may potentially contribute to the development of pulmonary infections in patients with severe myelitis (13, 14).

The aim of this study was to investigate the predictive factors for pulmonary infection in patients with severe myelitis, construct a prediction model by drawing a nomogram, and test the predictive power and clinical applicability of this model, providing a reference and direction for clinicians’ early assessment and proactive intervention.

## Methods

### Research subjects

**Figure 1:**
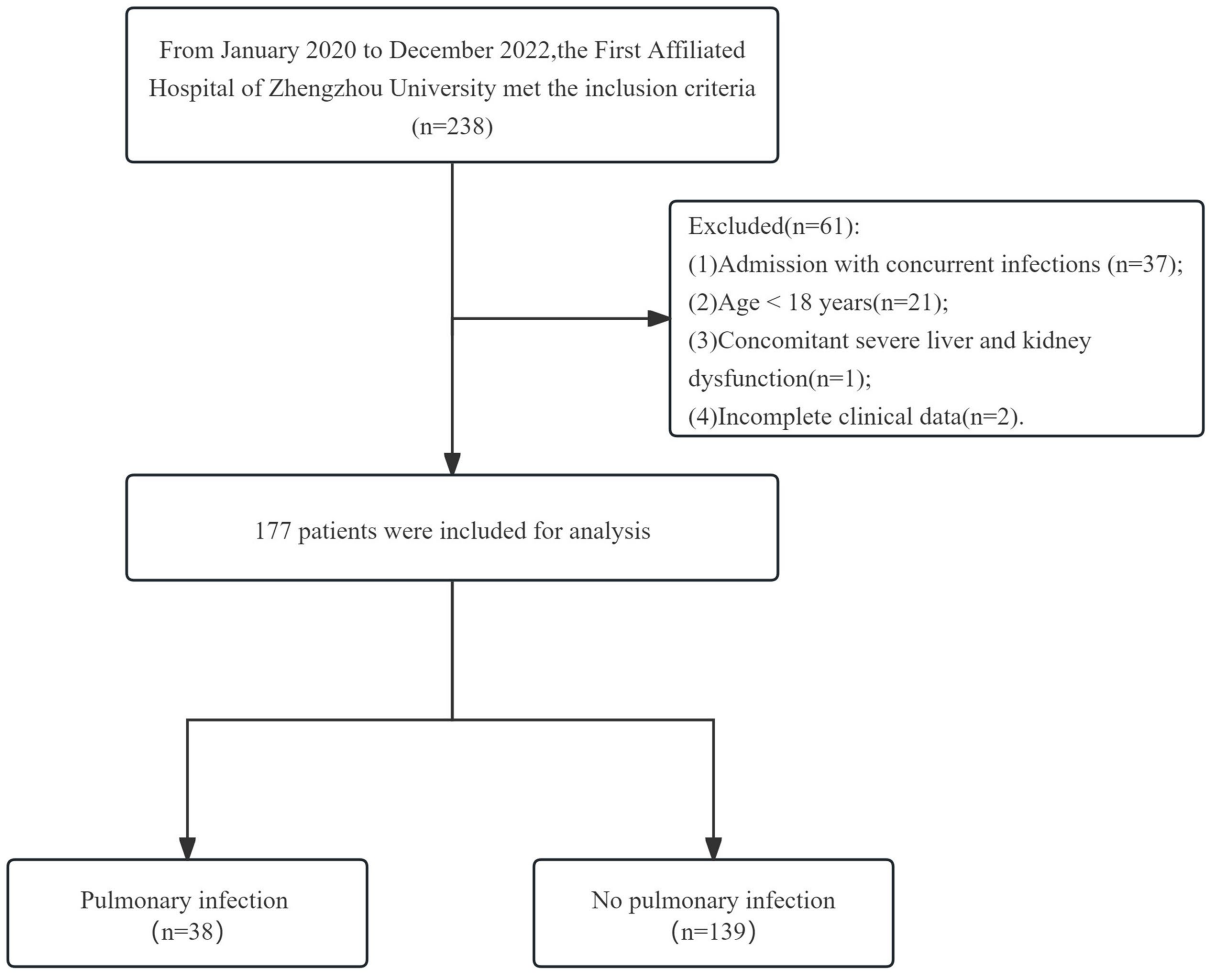
Selection process of study population.

This study retrospectively collected the clinical data of patients with severe myelitis from January 2020 to December 2022 in the First Affiliated Hospital of Zhengzhou University. Myelitis in this study included three types: acute myelitis, acute transverse myelitis and acute non-transverse myelitis. All patients met the diagnostic criteria for myelitis and were diagnosed by experienced neurologists. Inclusion criteria were: (1) meeting the diagnostic criteria for acute myelitis (15); (2) critically ill patients [Expanded Disability Status Scale (EDSS) score ≥ 6.0]. Exclusion criteria were: (1) admission with concurrent infections (37 patients); (2) Age < 18 years (21 patients); (3) concomitant severe liver or kidney dysfunction (1 patients); and (4) incomplete clinical data (2 patients). A total of 177 patients were included in the study, 38 patients with pulmonary infection and 139 patients without pulmonary infection (Figure 1). This study was approved by the Ethics Committee of the First Affiliated Hospital of Zhengzhou University (Number: 2023-KY-0409-002).

### Data collection

Demographic and clinical data were recorded for all patients, including gender, age, medical history, laboratory test, EDSS scores, and imaging findings. The severity of myelitis was assessed using EDSS, with EDSS score ≥ 6.0 defined as severe (16). According to the clinical diagnostic criteria for pulmonary infections formulated by the Respiratory Diseases Branch of the Chinese Medical Association (2018 edition), the occurrence of pulmonary infections during hospitalization was evaluated and determined for the included analysis of severe myelitis patients. The laboratory examination was performed within 48 h after admission.

### Statistical analysis

Normally distributed measurement data are presented as mean ± standard deviation (SD), and independent sample *t*-test; non-normally distributed continuous variables are presented as the median (IQR: interquartile range) was expressed and compared between groups using the Mann–Whitney *U* test. Categorical data were presented as the number of cases (percentage) and were compared using chi-squared tests. Univariate and multivariate logistic regression analysis were used to identify potential and independent predictors of pulmonary infection in severe myelitis, respectively. Collinearity diagnosis was performed for all variables included in the multivariate logistic regression. A nomogram was constructed based on the results of multivariate logistic regression analysis, and the predictive value and clinical applicability of the model were evaluated using the C-index, receiver operating characteristic (ROC) curve, calibration curve, Hosmer-Lemeshow goodness-of-fit test, and decision curve. Results with *p* < 0.05 were considered statistically significant. Statistical analyses were performed using SPSS 25.0, GraphPad Prism 9, and R software.

## Results

### Baseline characteristics and univariate analysis

Of the 177 severe myelitis patients, 58 (32.8%) were male and 119 (67.2%) were female, with a median age of 55 years. Among them, 38 (21.5%) had pulmonary infections and 139 (78.5%) had no pulmonary infections (Table 1).

**Table 1:** Comparison of clinical data between two groups of patients with severe myelitis.

	No pulmonary infection (*n* = 139)	Pulmonary infection (*n* = 38)	Z/χ^2^/t	*p* value
Demographic data				
Age, years, median (IQR)	56.00 (42.00, 66.00)	55.00 (41.50, 69.25)	−0.886	0.375
Male, n (%)	42 (30.2)	16 (42.1)	1.915	0.166
Onset time, days, median (IQR)	7.00 (3.00, 15.00)	6.00 (2.50, 15.00)	−0.901	0.367
Hospital stays, days, median (IQR)	18.00 (14.00–22.00)	18.50 (13.75–37.25)	−1.084	0.278
Past history				
Hypertension, n (%)	34 (24.5)	11 (28.9)	0.317	0.573
Diabetes, n (%)	22 (15.8)	6 (15.8)	0.000	0.995
Smoking, n (%)	16 (11.5)	7 (18.4)	1.260	0.262
Alcohol, n (%)	11 (7.9)	4 (10.5)	0.263	0.608
MRI				
High cervical cord lesion, n (%)			6.509	0.013
Yes	46 (33.1)	21 (55.3)		
No	93 (66.9)	17 (44.7)		
Extensive longitudinal myelopathy, n (%)				
>3 vertebral segments	79 (56.8)	26 (68.4)	1.660	0.198
Laboratory tests				
White blood cell, ×10^9^/L, median (IQR)	6.85 (5.49, 9.50)	8.96 (6.49, 11.74)	−2.842	0.004
Red blood cell, × 10^12^/L, mean ± SD	4.27 ± 0.49	4.15 ± 0.48	−1.387	0.167
Hemoglobin, g/L, mean ± SD	129.54 ± 15.84	126.50 ± 17.03	−1.031	0.304
Monocyte, ×10^9^/L, median (IQR)	0.42 (0.32, 0.58)	0.46 (0.29, 0.78)	−1.029	0.303
NLR, median (IQR)	2.94 (2.06, 5.27)	7.14 (3.63, 12.32)	−5.304	<0.001
PLR, median (IQR)	159.82 (113.88, 225.27)	201.52 (136.45, 293.39)	−2.379	0.017
NPAR, median (IQR)	1.70 (1.48, 1.92)	2.03 (1.74, 2.38)	−4.653	<0.001
Total protein, g/L, mean ± SD	66.73 ± 7.07	66.07 ± 7.46	−0.504	0.615
Globulin, g/L, median (IQR)	25.30 (22.40, 28.70)	26.10 (22.48–31.18)	−0.656	0.512
Alanine aminotransferase, U/L, median (IQR)	17.00 (12.00, 26.00)	20.50 (12.50, 34.75)	−1.019	0.308
Aspartate aminotransferase, U/L, median (IQR)	19.00 (14.00, 24.00)	20.50 (15.75, 28.25)	−1.128	0.259
Urea, mmol/L, median (IQR)	5.40 (4.20, 6.80)	5.75 (4.43, 8.15)	−1.092	0.275
Creatinine, umol/L, median (IQR)	56.00 (48.00, 63.00)	56.00 (49.50, 73.25)	−0.91	0.363
Uric Acid, umol/L, median (IQR)	230.00 (181.00, 281.00)	225.00 (175.75, 281.75)	−0.238	0.812
Prothrombin time, s, median (IQR)	10.60 (10.20, 11.20)	10.95 (10.38, 11.33)	−1.573	0.116
Prothrombin time activity, %, median (IQR)	106.00 (96.50, 112.30)	101.00 (96.75, 109.25)	−1.340	0.180
International normalized ratio, median (IQR)	0.96 (0.91, 1.03)	0.99 (0.95, 1.02)	−1.729	0.084
Activated partial thromboplastin time, s, median (IQR)	27.17 (24.70, 29.20)	27.65 (26.23, 29.35)	−1.215	0.224
Determination of fibrinogen, g/L, median (IQR)	2.93 (2.46, 3.52)	2.90 (2.43, 3.42)	−0.330	0.741
Thrombin time, s, median (IQR)	15.70 (14.40, 17.50)	15.15 (13.73, 17.01)	−1.671	0.095
D-dimer, g/L, median (IQR)	0.32 (0.15, 0.85)	0.32 (0.15, 0.75)	−0.127	0.899
Cerebrospinal fluid tests (*n* = 141)				
Albumin quotient, ×10^−3^, median (IQR)	6.76 (5.01, 10.03)	5.75 (4.27, 9.78)	−1.454	0.146
Immunoglobulin quotient, ×10^−3^, median (IQR)	4.93 (3.21, 8.03)	4.59 (2.75, 6.60)	−1.137	0.256
IgG index, median (IQR)	0.72 (0.62, 0.89)	0.69 (0.58, 0.87)	−0.373	0.709
IgG SR, mg/24 h, median (IQR)	6.20 (0.86, 15.99)	5.53 (1.13, 10.46)	−1.422	0.155
Thyroid function (*n* = 150)				
FT3, pmol/L, mean ± SD	4.28 ± 0.71	4.04 ± 0.71	−1.599	0.112
FT4, pmol/L, median (IQR)	12.60 (10.91, 14.64)	12.42 (10.34, 13.92)	−0.336	0.737
TSH, uIU/mL, median (IQR)	1.53 (0.81, 3.12)	1.17 (0.50, 2.45)	−0.992	0.321

IQR, interquartile range; SD, standard deviation; MRI, magnetic resonance imaging; High cervical cord lesions: C5 and above spinal cord segment lesions; NLR, neutrophil/lymphocyte ratio; PLR, platelet/lymphocyte ratio; NPAR, neutrophil percentage/albumin ratio; IgG, immunoglobulin G; SR, synthesis rate; FT3, free triiodothyronine; FT4, free thyroxine; TSH, thyroid stimulating hormone.

**Figure 2:**
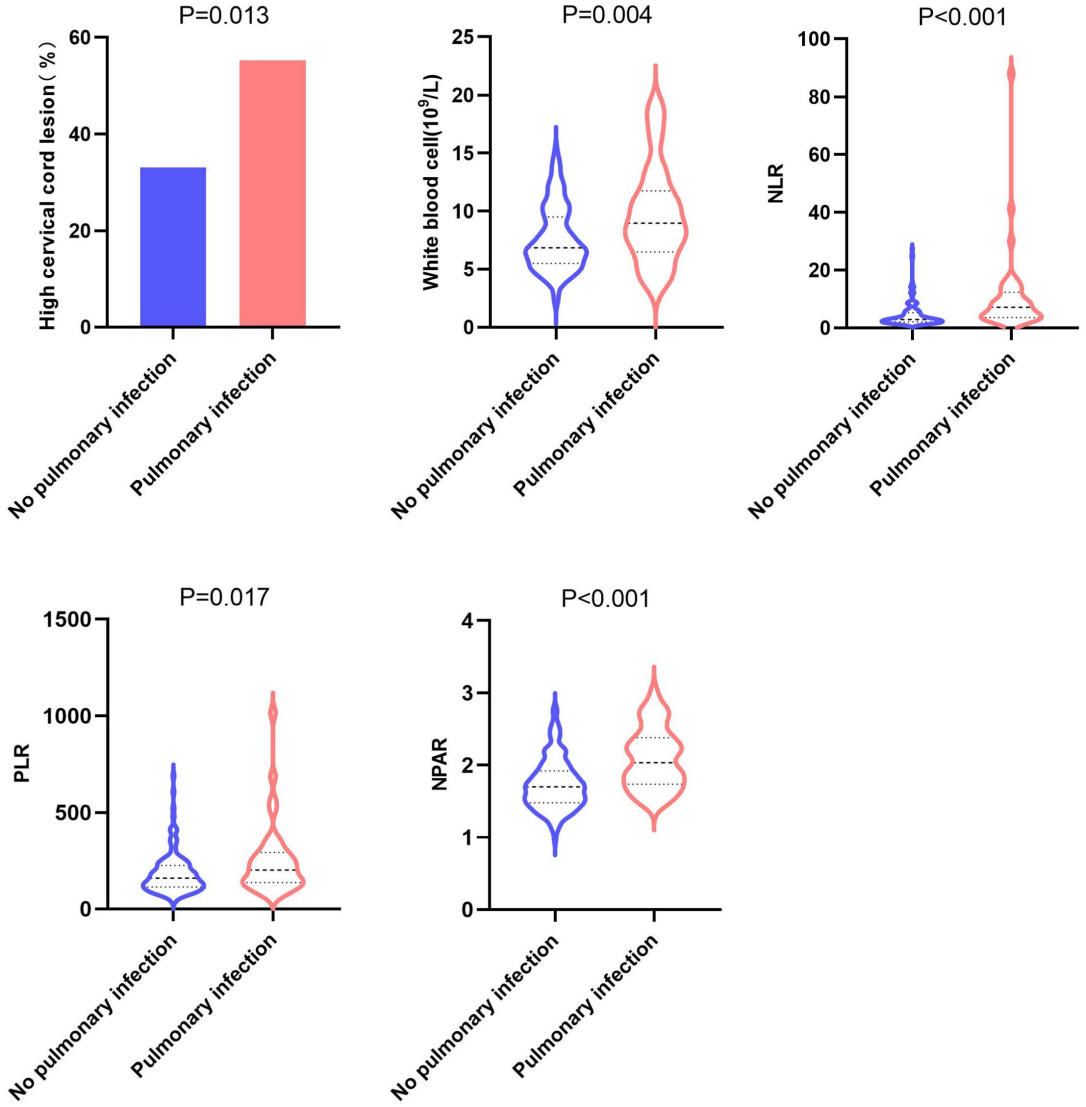
Potential influencing factors pulmonary infection in patients with severe myelitis.

The results of univariate analysis showed that compared to the group without pulmonary infections, the group with pulmonary infections had a higher proportion of patients with high cervical cord lesions (C5 and above spinal cord segment lesions), higher white blood cell count, higher neutrophil-to-lymphocyte ratio (NLR), higher platelet-to-lymphocyte ratio (PLR), and higher neutrophil percentage-to-albumin ratio (NPAR). The differences were statistically significant (*p* < 0.05) (Table 1 and Figure 2).

### Multivariate logistic regression analysis of risk factors for pulmonary infection

**Figure 3:**
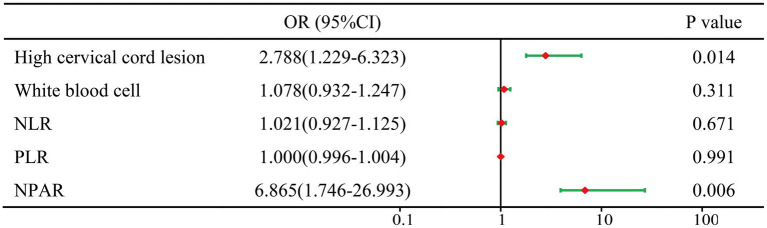
Forest plots of multivariable logistic regression affecting pulmonary infection. OR, odds ratio; CI, confidence interval; high cervical spinal cord lesions: C5 and above spinal cord segmental lesions; NLR, neutrophil/lymphocyte ratio; PLR, platelet/lymphocyte ratio; NPAR, neutrophil percentage/albumin ratio.

In order to screen the independent influencing factors of pulmonary infection in patients with severe myelitis, variables with *p* < 0.05 from the univariate analysis were included in multivariate logistic regression analysis, as shown in Figure 3. The collinearity test showed that variance inflation factor for all variables was below 2.3, tolerance was greater than 0.4, indicating no collinearity among the variables. Multivariate logistic regression analysis showed NPAR (*OR* = 6.865, 95% *CI*: 1.746–26.993, *p* = 0.006) and high cervical cord lesions (*OR* = 2.788, 95% *CI*: 1.229–6.323, *p* = 0.014) were independent influencing factor for the occurrence of pulmonary infections in patients.

### Nomogram model construction and validation

**Figure 4:**
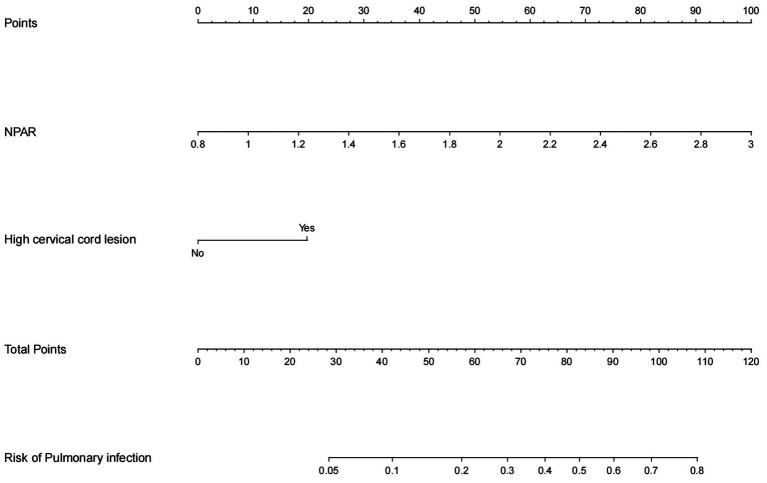
Nomogram predicting pulmonary infection in patients with severe myelitis.

**Figure 5:**
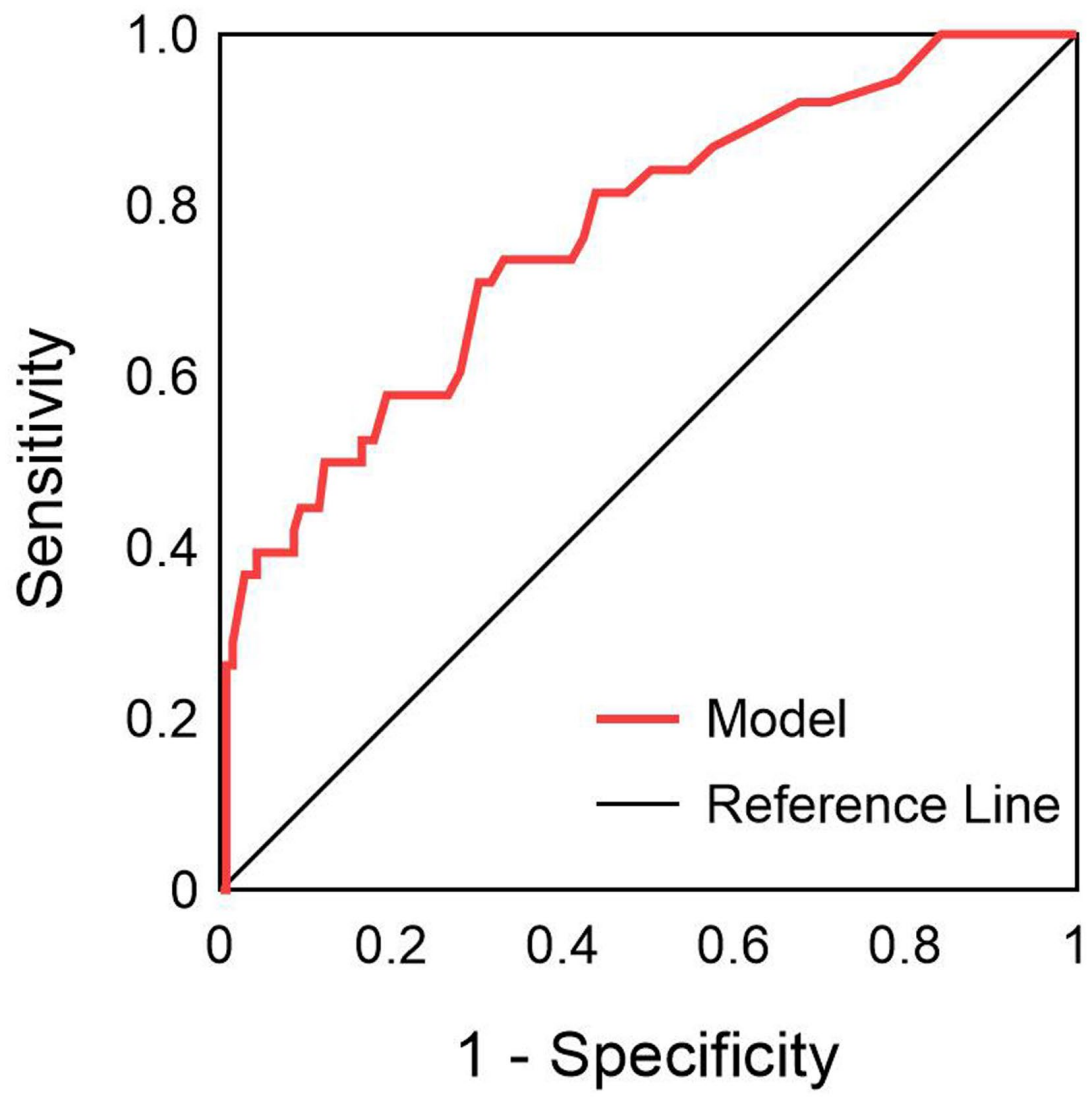
ROC curve of NPAR combined with high cervical myelopathy in predicting pulmonary infection in patients with severe myelitis.

Based on the results of multivariate logistic regression analysis, a nomogram model was constructed to predict the risk of pulmonary infections in severe myelitis (Figure 4). The C-index was 0.766 (95% *CI*, 0.678–0.854). The ROC curve analysis (Figure 5) showed an area under the curve (AUC) of 0.766 (95% *CI*, 0.678–0.853, *p* < 0.001), with a sensitivity of 71% and specificity of 70%. These results suggest that NPAR and high cervical cord lesions has a good predictive performance for the occurrence of pulmonary infections in patients with severe myelitis.

**Figure 6:**
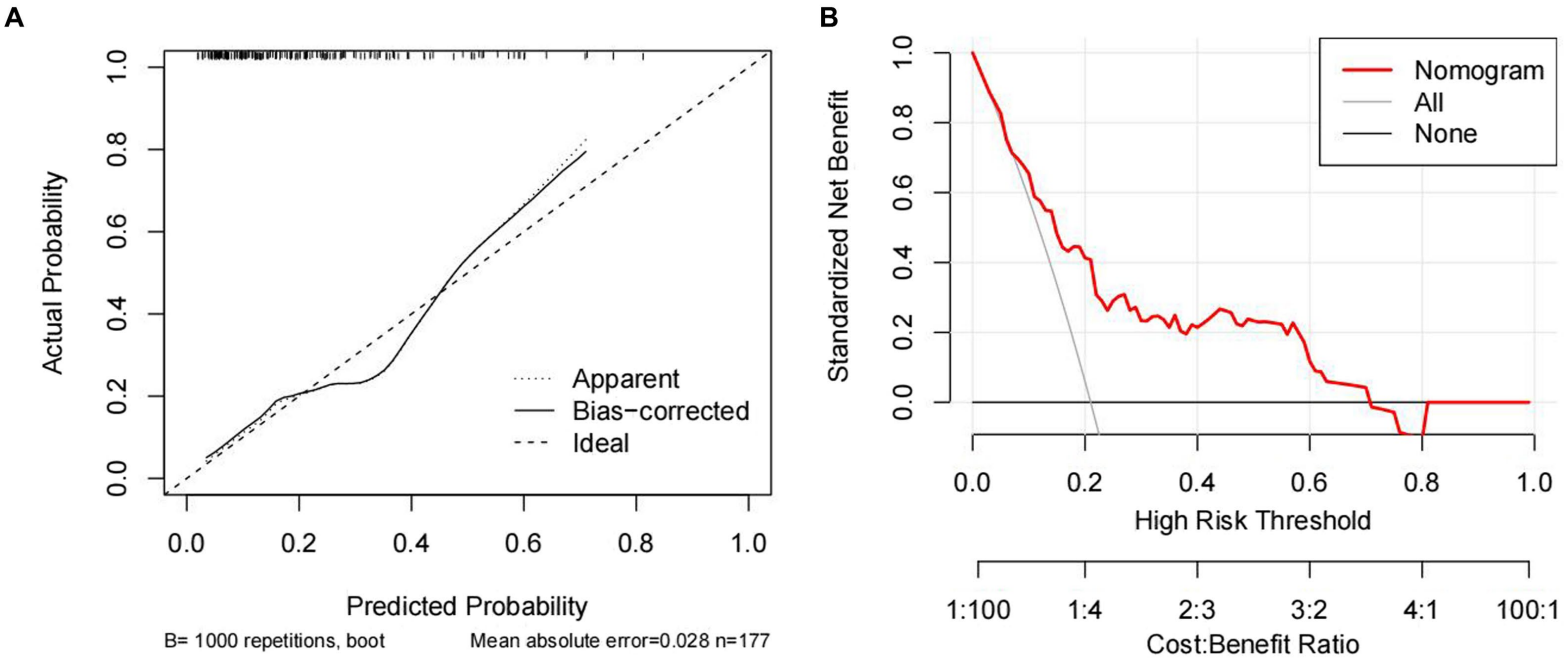
Calibration curve **(A)** and decision curve analysis **(B)** for predicting pulmonary infection in patients with severe myelitis.

The nomogram model was validated by bootstrap, 1,000 cycles of testing were performed, the calibration curve (Figure 6A) and the Hosmer-Lemeshow goodness-of-fit test (*χ*^2^ = 9.539, *p* = 0.299) the results showed that the nomogram model predicted the occurrence of pulmonary infection in good agreement with the actual situation, and the model had excellent predictive accuracy. Additionally, the decision curve analysis (Figure 6B) revealed good clinical applicability and validity of the model.

## Discussion

In this study, we performed the first early prediction of concurrent pulmonary infection in patients with severe myelitis by admission indicators. The results showed that NPAR and high cervical cord lesions were independent risk factors for the development of pulmonary infection in patients with severe myelitis. A nomogram model was constructed based on two variables, NPAR and high cervical spinal cord lesions. C-index, calibration curve, Hosmer-Lemeshow goodness-of-fit test, and decision curve analysis confirmed that the prediction model has good consistency and clinical applicability. Furthermore, the ROC results indicated that the combination of NPAR and high cervical cord lesions had higher accuracy in evaluating the occurrence of pulmonary infections in patients. These findings will guide clinicians in their stratified management and early intervention for similar patients to improve their prognosis.

NPAR is a novel biomarker that has received increasing attention in recent years, which combines neutrophils percentage and albumin to more comprehensively reflect the body’s status in terms of inflammation, immunity, and nutrition. It has been used to assess disease severity, prognosis, and infectious complications in various of diseases (9, 17, 18). In the acute phase of severe myelitis, a large number of inflammatory mediators are released, leading to neutrophil recruitment and activation, further amplifying the inflammatory response. As the most abundant leukocytes in blood, neutrophils are the first line of defense against invading pathogens and play a crucial role in innate immune responses (19). Neutrophils exhibit chemotaxis toward immune cells, phagocytosis of pathogens, and disruption of immune tolerance. They also participate in establishing the inflammatory environment by producing and releasing reactive oxygen species, cytokines, proteases, and other tissue-damaging molecules, while modulating adaptive immunity. When the neutrophil percentage increases, the body inflammation increases and immune imbalance, and susceptibility to infection increases (20).

Accumulated evidence suggests that the presence of an inflammatory response during the acute phase of the disease can significantly impact the nutritional status of the body, leading to hypoproteinemia (21). Albumin is the main protein in human plasma and plays various physiological functions including maintaining osmotic pressure, binding plasma molecules, detoxifying, immune and inflammatory regulation (22, 23). Studies have found that albumin not only inhibits neutrophil migration but also reduces cytokine production, thereby exerting anti-inflammatory effects (24, 25). Given the important role of albumin in reflecting the body’s nutritional status, decreased levels may lead to impaired immune function and increase the risk of infection (26). Additionally, a retrospective study has also demonstrated that low albumin levels increase the risk of central nervous system infections and mortality (27).

The pulmonary infection in patients with severe cervical spinal cord lesions may be related to the following reasons. Firstly, the phrenic nerve is composed of the anterior branches of the third, fourth, and fifth pairs of cervical nerves. When the C3-C5 spinal cord is injured, the diaphragm muscle innervated by the phrenic nerve becomes paralyzed. As the major inspiratory muscle, its impairment can partially or completely restrict the inspiratory function, leading to ventilation disorders and, in severe cases, even suffocation. Secondly, in high cervical spinal cord injury, the sympathetic innervation to the lungs is weakened while the activity of the vagus nerve is enhanced, resulting in decreased levels of circulating adrenaline and increased airway resistance, leading to airway narrowing (28). Thirdly, with high cervical cord lesions, the pathway from the brainstem respiratory center to the respiratory motor neurons in the cervical and thoracic regions of the spinal cord is blocked, causing respiratory muscle paralysis. This leads to a decrease in respiratory capacity and clearance ability of the airways, increasing the risk of ineffective coughing, mucus retention, and atelectasis (29). Finally, high cervical cord injury causes limb paralysis, reduces chest wall compliance, increases abdominal wall compliance, and results in increased chest stiffness and respiratory effort. Therefore, high cervical cord lesions are closely associated with the occurrence of pulmonary infection. In clinical practice, for patients with high cervical cord disease, attention should be paid to respiratory management to reduce the risk of pulmonary infection and thus improve the prognosis of patients.

Due to the severity of their condition and long-term bed rest, patients with severe myelitis are extremely susceptible to pulmonary infections. The occurrence of pulmonary infections will aggravate the patient’s condition, increase the difficulty of treatment, prolong hospitalization time, and seriously affect the patient’s prognosis and mortality (30, 31). Therefore, early screening of possible populations with pulmonary infections is crucial for improving the prognosis of myelitis and reducing mortality. A nomogram model based on the combination of NPAR and high cervical myelopathy exhibits favorable predictive performance and clinical applicability, which can not only help clinicians to early predict the occurrence of pulmonary infection in patients and perform stratified management, but also guide clinicians to determine the focus and direction of intervention to reduce the risk of pulmonary infection in patients.

This study has certain limitations. Firstly, it is a single-center, retrospective study, which may introduce selection bias. Additionally, due to limited data availability, external validation was not conducted. Furthermore, there are some shortcomings in the model, which may be related to some influencing factors are not included and require further study.

In conclusion, we found that NPAR and high cervical cord lesions are closely associated with the occurrence of pulmonary infections in patients with severe myelitis. The nomogram model combining the two can efficiently and reliably predict the occurrence of pulmonary infection in patients with severe myelitis, which may provide reference and inspiration for further studies.

## Data availability statement

The raw data supporting the conclusions of this article will be made available by the authors, without undue reservation.

## Ethics statement

The studies involving humans were approved by the Ethics Committee of the First Affiliated Hospital of Zhengzhou University. The studies were conducted in accordance with the local legislation and institutional requirements. The ethics committee/institutional review board waived the requirement of written informed consent for participation from the participants or the participants’ legal guardians/next of kin because written informed consent from the participants’ legal guardian/next of kin was not required to participate in this study in accordance with the national legislation and the institutional requirements.

## Author contributions

FY: Formal analysis, Methodology, Software, Writing – original draft, Writing – review & editing. RD: Data curation, Writing – review & editing. YW: Data curation, Writing – review & editing. JG: Data curation, Writing – review & editing. QZ: Data curation, Writing – review & editing. LW: Data curation, Writing – review & editing. PH: Writing – review & editing. JQ: Writing – review & editing. DS: Data curation, Writing – review & editing. ZR: Data curation, Writing – review & editing. JT: Project administration, Writing – review & editing. WM: Funding acquisition, Project administration, Supervision, Writing – review & editing.
